# Automated tools for systematic review screening methods: an application of machine learning for sexual orientation and gender identity measurement in health research

**DOI:** 10.5195/jmla.2025.1860

**Published:** 2025-01-14

**Authors:** Ashleigh J. Rich, Emma L. McGorray, Carrie Baldwin-SoRelle, Michelle Cawley, Karen Grigg, Lauren B. Beach, Gregory Phillips, Tonia Poteat

**Affiliations:** 1 Ashleigh.rich@duke.edu, School of Nursing, Duke University, Durham, NC; 2 EmmaMcgorray2023@u.northwestern.edu, Department of Psychology, Northwestern University, Evanston, IL; 3 chbs@email.unc.edu, Health Sciences Library, University of North Carolina Chapel Hill, Chapel Hill, NC; 4 mcawley@email.unc.edu, Health Sciences Library, University of North Carolina Chapel Hill, Chapel Hill, NC; 5 kgrigg@email.unc.edu, Health Sciences Library, University of North Carolina Chapel Hill, Chapel Hill, NC; 6 lauren.beach@northwestern.edu, Department of Medical Social Sciences, Department of Preventative Medicine, Northwestern University, Evanston, IL; 7 Glp2@northwestern.edu, Department of Medical Social Sciences, Department of Preventative Medicine, Northwestern University, Evanston, IL; 8 tonia.poteat@duke.edu, School of Nursing, Duke University, Durham, NC

**Keywords:** Sexual and Gender Minorities, Health, Methods, Systematic Review, Automation

## Abstract

**Objective:**

Sexual and gender minority (SGM) populations experience health disparities compared to heterosexual and cisgender populations. The development of accurate, comprehensive sexual orientation and gender identity (SOGI) measures is fundamental to quantify and address SGM disparities, which first requires identifying SOGI-related research. As part of a larger project reviewing and synthesizing how SOGI has been assessed within the health literature, we provide an example of the application of automated tools for systematic reviews to the area of SOGI measurement.

**Methods:**

In collaboration with research librarians, a three-phase approach was used to prioritize screening for a set of 11,441 SOGI measurement studies published since 2012. In Phase 1, search results were stratified into two groups (title with vs. without measurement-related terms); titles with measurement-related terms were manually screened. In Phase 2, supervised clustering using DoCTER software was used to sort the remaining studies based on relevance. In Phase 3, supervised machine learning using DoCTER was used to further identify which studies deemed low relevance in Phase 2 should be prioritized for manual screening.

**Results:**

1,607 studies were identified in Phase 1. Across Phases 2 and 3, the research team excluded 5,056 of the remaining 9,834 studies using DoCTER. In manual review, the percentage of relevant studies in results screened manually was low, ranging from 0.1 to 7.8 percent.

**Conclusions:**

Automated tools used in collaboration with research librarians have the potential to save hundreds of hours of human labor in large-scale systematic reviews of SGM health research.

## INTRODUCTION

Sexual and gender minority (SGM) populations disproportionately experience poor health compared to heterosexual and cisgender populations. For example, SGM populations experience increased risk for physical and mental health issues such as depression, anxiety, HIV, and some cancers [1, 2], with research suggesting that these disparities are related to experiences of minority stress (e.g., stigmatization, discrimination, negative internalized attitudes) in relation to one's SGM identity [3, 4]. While existing research makes clear that these disparities exist, understanding the extent and nature of these disparities requires comprehensive, accurate measurement of sexual orientation and gender identity (SOGI). Accurate and consistent measurement of SOGI helps researchers to paint the clearest picture of the health inequities faced by SGM populations. Advancing this understanding is necessary to develop interventions to promote SGM health equity.

Existing SOGI measurement strategies often fall short of providing the information needed to fully understand SGM disparities. One issue is a lack of standardized validated measurement across health research and practice contexts, which prevents straightforward integration of findings from different settings. Existing measurement approaches often do not capture the multidimensionality of sexual orientation, a construct that includes attraction, behavior, and identity [5]. Sex and gender are often conflated, captured in a limited capacity via one step item (i.e. ‘male’, ‘female’, ‘transgender’) instead of best practice two-step measures (i.e. a sex assigned at birth item plus a current gender identity item) [6]. Further, gender and sex are often treated as binary constructs encompassing only identities such as “man” and “woman” or “male” and “female,” reinforcing notions of gender and sex that prevent nonbinary and intersex identities from being appropriately measured [7]. The lack of pre-existing sampling frames as well as the historical exclusion of SGM people from routine public health surveillance and other health research efforts constitute other challenges [8].

Even ongoing efforts to address these inconsistencies and offer recommendations for standardized SOGI measurement can replicate limitations of prevailing measurement strategies. Importantly, the recently released US National Academies of Science, Engineering, and Medicine (NASEM) landmark 2022 report, *Measuring Sex, Gender Identity and Sexual Orientation* [9], systematically evaluating SSOGI measurement in the US, providing measurement guidance, and setting related research priorities for the NIH and beyond, is limited by gender identity measurement recommendations that may conflate sex and gender and erase non-binary identities as well as fail to capture sexual orientation multidimensionality. To better understand these issues and get a comprehensive view of measurement of SOGI in health research, we undertook a systematic review. Conducting a systematic review in SGM health poses a number of challenges. First, opportunities for SGM health research are growing [10], producing a large body of research results to screen when conducting systematic reviews. Second, searching for research related to SOGI measurement involves key terms likely to be found in a wide range of studies, including studies completely unrelated to SGM health or SOGI measurement. This means that searching for research in this area is likely to produce a large amount of research irrelevant to researchers' questions, increasing the time needed to screen search results.

One potential solution to this problem is the use of automated tools such as machine learning, which have long been used to minimize the time and labor needed to screen the large volume of search results that arises when investigating complicated or wide-ranging research questions [11]. However, despite these tools' potential, [12–18], they have not often been leveraged to streamline the process of conducting systematic reviews [11].

Unfamiliarity with machine learning and other automated tools may be one barrier to implementation of these tools in systematic reviews. However, librarians have access to the training, expertise, and software needed to conduct effective searches and screen results using automated tools [11]. Collaborations with librarians trained in automation tools pose a promising opportunity for research teams to effectively use these tools to ensure high-quality, efficient reviews, and we established such a collaboration in the current research. As part of a larger project reviewing and synthesizing how SOGI has been assessed within the health literature, we provide an example of the application of automated tools for systematic reviews to the area of SOGI measurement.

## METHODS

### Team Roles

The University of North Carolina Health Sciences Library (UNC HSL) offers both consulting and co-authoring services to affiliated researchers. As co-authors, librarians lead the construction of search strategies, perform the searches, advise on automation tools, maintain an EndNote Library, set up the review within Covidence, and contribute to the manuscript. The non-librarian researchers co-design and review the search strategy, screen the studies in both the title/abstract and full text stages, assess quality of included studies, synthesize research, and write the review.

### Search Methods

The search strategy, developed by the research team and librarians, included controlled vocabulary terms and keywords based on the concepts of a) sexual and gender minorities (e.g., gay, lesbian, bisexual, transgender) and b) measurement (**Table 1**). Health sciences librarians conducted comprehensive searches in four bibliographic databases: PubMed (NLM), CINAHL (EBSCOhost), PsycInfo (EBSCOhost), and Health and Psychosocial Instruments-HAPI (EBSCOhost). Based on the volume of the results, availability of potential databases, and the indexing of the known journals of interest, the team selected subject-specific databases that would be most likely to contain relevant results. The search was limited to English-language documents with a published date of 2012 or later. Since the field of SGM health research has exploded in the past decade, SGM literature reviews with longer timeframes ultimately include research since 2010 [19], and SOGI measurement prior to the recent past likely includes discredited findings, the team applied a date filter to focus on the state of the SOGI literature in the past decade. The search included peer-reviewed journal articles reporting primary data focused on SSOGI measurement in health research, conducted in the United States. Conference abstracts, case reports, editorials, reviews, and any other non-peer reviewed literature were excluded from eligibility.

**Table 1:** Measurement-related terms searched in title used to stratify search results.

Root term searched in title field	Terms captured
Instrum^*^	Instrument; Instrumental; Instruments; Instrumentation
Measur^*^	Measure; Measures; Measured; Measurement; Measurements; Measuring
Seal^*^	Scale; Scales; Scaled; Scaling
Surv^*^	Survey; Surveyed; Surveys; Surveying; Surveil; Surveillance; Surveillmg
Valid^*^	Valid; Validate; Validates; Validated; Validating; Validation; Validity

* indicates truncation to capture alternate word forms.

### Prioritization of Literature for Manual Screening

**Figure 1:**
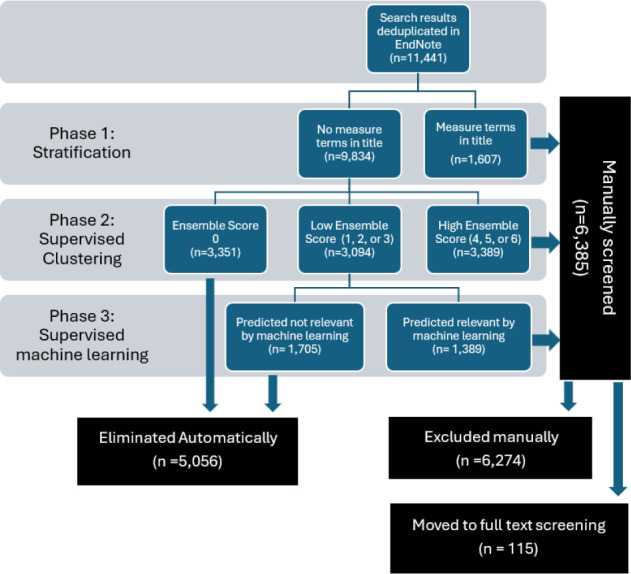
Summary of screening methodology by phase

Studies most likely to be relevant from the search results were prioritized for manual screening in three phases described below (**Figure 1)**. Citations were then manually screened for inclusion at the title and abstract level, then at the full text level, by two independent subject matter experts using Covidence Systematic Review Software [20].

#### Phase 1: Stratification

Following de-duplication in EndNote, the search results were stratified into two groups. Studies with one or more measurement-related terms in the title (**Table 1**) were identified in EndNote and screened manually in Phase 1 and the remaining studies moved forward to Phase 2. The team chose to stratify the results because we expected that studies with measurement-related terms in the title were more likely to make measurement a focus of the paper, rather than an incidental mention in the abstract. Cawley noted that using a stratified approach can be helpful to ensure a subset of results are all considered in manual review before the application of automation tools such as machine learning [11].

#### Phase 2: Supervised Clustering Using DoCTER

In Phases 2 and 3, results not screened in Phase 1 were prioritized with Document Classification and Topic Extraction Resource (DoCTER) [21]. DoCTER uses publicly available clustering and machine learning algorithms to prioritize search results using the text of titles and abstracts, including K-means, non-negative matrix factorization (NMF), Naïve Bayes, linear support vector machines (linear SVC), and k-nearest neighbor (KNN). Varghese et al. provides details on these conventional machine learning algorithms as used by DoCTER [22].

In Phase 2, supervised clustering—a form of semi-supervised learning that groups an unclassified corpus of studies and a set of known relevant (i.e., “seed”) studies into clusters based on text similarities in titles and abstracts—was used. Seed studies are a form of training data but require fewer positive studies than typically necessary for machine learning algorithms. Ideally, a target of 25–50 seeds should be identified by reviewing a random subset of search results.

Clusters containing seed studies are likely to contain relevant unclassified studies. Clusters are prioritized for manual screening based on the number of seed studies they contain until a desired recall target is reached. For example, if 100 seed studies are used and 95% recall is desired, then clusters are prioritized for manual review until 95 or more of the seeds are captured.

Seeds (positive training data) should be identified at random from the unclassified corpus to avoid selection bias and to produce accurate predictions of recall. Ideally, subject matter experts should screen studies at random to select at least 25 seeds. Negative training data are not necessary for supervised clustering. Varghese, Cawley, and Hong provide further details on supervised clustering and demonstrate that the method rivals accuracy rates of supervised machine learning algorithms while requiring less training data [22]. Cawley provides summary data for a series of case studies using the approaches outlined here by librarians at UNC HSL including stratification and prioritizing studies for screening in a two-phased approach with supervised clustering and supervised machine learning [11].

The ensemble approach to supervised clustering uses two algorithms: k-means and nonnegative matrix factorization (NMF) and three cluster sizes: 10, 20, and 30. Using each algorithm with the three different cluster numbers yields six different clustering models (e.g., KM-10 model is the k-means algorithm with 10 clusters and KM-20 is the k-means algorithm with 20 clusters). The six models were applied to title and abstract text of the citations not screened in Phase 1, along with a set of seed studies.

The output of supervised clustering with a six-model ensemble approach is an ensemble score (ES) for each study that ranges from 6 to 0. The ES indicates the number of models where the study was found in a cluster prioritized by DoCTER. Citations with ES = 6 are predicted to have a higher likelihood of relevance compared to studies with lower ensemble scores. Citations with an ES = 0 are not predicted relevant by any of the six models and are typically excluded without manual screening.

#### Phase 3: Supervised Machine Learning Using DoCTER

In Phase 3, results less likely to be relevant (ES = 3, 2, or 1) from Phase 2 were further prioritized using supervised machine learning. The decision to move to supervised machine learning is recommended when precision (i.e., the number of relevant studies as a percentage of all studies screened manually) starts to diminish rapidly. Moving to machine learning to prioritize studies further allows for more studies to be excluded without manual screening.

Supervised machine learning uses different algorithms than clustering (e.g., naïve Bayes, support vector machines) and requires a relatively large training dataset. Whereas supervised clustering requires approximately 25–50 relevant studies for training data, machine learning requires positive and negative training data. The amount of training data needed varies based on many factors but from experience we endeavor to use at least 100 positive studies. The sizes of training datasets used for this approach range from the low hundreds (van de Bulk et al.) to high thousands (Liao et al.). Cawley et al. ran three simulations of a similar application of machine learning and used approximately 200 positive studies for training data in each of the three simulations and reached 95% recall in each instance [23, 24].

After running the supervised machine learning process in DoCTER, each study is given a probability score based on how likely it is to be relevant. Unlike supervised clustering with an ensemble approach, which puts studies into batches, machine learning algorithms provide a probability score for each individual study. The training data for supervised machine learning were derived from studies manually screened in Phases 1 and 2. Cawley provides evidence that a two-step approach of supervised clustering followed by supervised machine learning is effective at reducing the manual screening burden without significantly impacting recall of relevant articles and that training data for supervised machine learning can be drawn from labelled data in earlier steps [11].

## RESULTS

### Search Results

In total, 17,814 citations were returned from all databases searched. Results were imported to EndNote and duplicates were removed. After removing duplicates, 11,441 citations were prioritized for manual screening.

### Phase 1 Results

Phase 1 included all results with measurement-related terms in title (**Table 1**), identified by a keyword search in EndNote. All 1,607 results in this group were screened manually, given that these studies had a higher likelihood of being relevant (**Figure 1**) and 85 relevant studies were identified during this step.

### Phase 2 Results

Studies not containing a measurement-related term in title (n = 9,834) were moved to Phase 2 and prioritized with DoCTER [21] software using supervised clustering with an ensemble approach (**Figure 1**).

Prior to Phase 1, the research team screened the titles and abstracts of 500 studies, selected at random from the search results, to identify seeds. As noted above, seeds should be identified from a random sample of the unclassified corpus to avoid selection bias and allow for accurate predictions of recall. In this step, 39 studies were classified as relevant by subject matter experts and used as seeds to prioritize the 9,834 results not screened in Phase 1.

**Figure 2:**
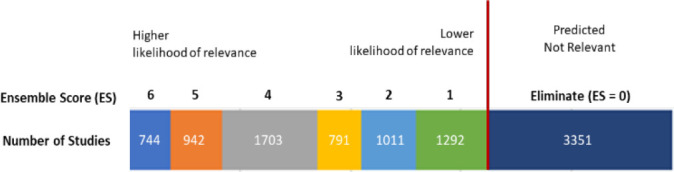
Supervised clustering results

Note: Studies with ensemble score (ES) of greater than 1 were considered for manual screening or further prioritization.

In Phase 2, supervised clustering with an ensemble approach was used to prioritize results for manual screening. In total, 6,483 results had an ES = 1 or higher and were retained for either manual screening or further prioritization. A total of 3,351 results had an ES = 0 (**Figure 2**) and were excluded without manual screening. For Phase 2 of screening, studies with an ES = 4 or higher were screened manually (n = 3,389) (**Figure 1**). Only 10 relevant studies were found in these results. This very low precision is unusual but was not unexpected by the research team. The nature of the systematic review question necessitated a broad search strategy that would result in a large number of false positives. Due to the very low precision for studies with ES = 6, 5, or 4, the research team further prioritized the remaining studies with an ES = 1, 2, or 3 in Phase 3 using supervised machine learning.

### Phase 3 Results

For Phase 3 of screening, supervised machine learning was applied to further prioritize studies with an ES = 3, 2, or 1 (n = 3,094). Training data for supervised machine learning were derived from screening results of Phases 1 and 2. The machine learning algorithm prioritized a total of 1,389 studies as likely to be relevant using a recall threshold of 95%. These studies were manually screened, and all remaining studies (n = 1,705) were excluded without manual review (Phase 3; **Figure 1**). Studies were screened in order of probability score in descending order from most likely to be relevant to least likely to be relevant.

Of the 1,389 studies screened manually in Phase 3, only 11 relevant studies were identified after full-text screening was completed (1 study was excluded after full text screening). The 11 studies identified in Phase 3 were found in the top 25% of 1,389 studies screened for Phase 3 when ordered by probability of being relevant. The bottom 75% of studies (n = 1,063) did not contain any relevant studies. This provides evidence that the approach was effective and that few, if any, additional relevant studies were likely to be found in the studies excluded without manual screening.

## DISCUSSION

In this study, a machine learning approach was applied to literature screening in the conduct of a systematic review of SOGI measurement research. This work provides a practical application of automated methods to systematic reviews in the context of SOGI measurement and SGM health, illustrating that automated tools can help researchers to efficiently use time and labor resources. Such considerations are especially important in fast-growing areas such as SGM health and SOGI measurement where low-precision searches will likely remain normative; this study serves as a potential model for researchers in these areas. Nearly all health domains have fast growing areas of research (e.g., emerging infectious diseases) or topics where the historical volume of literature consistently poses a challenge any time a new research question is asked in the domain (e.g., tobacco- and HIV-related research).

This project also illustrates the utility of collaborations between research teams and health science librarians when conducting systematic reviews, as librarians have training in the required skillset and access to the necessary software to implement automated tools [11], enabling research partners to focus on their disciplinary and content area expertise.

The application of machine learning to systematic literature reviews is most often for the literature screening step [25]. In this study, the research team screened a total of 6,385 studies manually. Using supervised clustering and supervised machine learning in Phases 2 and 3 allowed us to exclude 5,056 studies without manual screening.

At all phases of manual review, search precision was very low and ranged from 0.1 to 7.8 percent with the highest precision in Phase 1. Overall search precision was 1.8%, which was consistent with the research team's expectations of relatively high sensitivity and low specificity given the growing SGM health research literature and relatively sparse research in SOGI measurement. The risk of misclassification is low as SOGI terms (i.e. sexual orientation, gender identity) are very specific to SGM research and not used in other disciplines. Given the low search precision following manual screening for all three phases, studies with an ES = 0 were excluded from manual screening. Tran et al. note that reducing the number of citations that must be screened manually using automation may not be recommended for reviews assessing efficacy of clinical interventions but may be acceptable in other instances [26]. Further, it is notable that using automation to reduce the number of citations that must be screened manually may allow research teams to develop broader research questions and contribute to a paradigm shift in how relevant literature is found [24, 26].

Using machine learning to exclude studies without manual review carries the risk of Type 2 errors (i.e., false negatives). Saving time and resources is the tradeoff to missing relevant studies. Consensus is that a recall threshold of 95% is an acceptable level of risk for systematic reviews using AI-assisted screening methodology [27, 28]. DoCTER and other similar applications allow the user to specify the recall threshold which is estimated using training data. Given the statistical underpinnings of the stopping criteria, we are confident we missed 5% or fewer of the relevant studies [11].

When available, simulation data bears this out and we consistently find 95% or higher recall using this methodology on simulated data [11, 24]. With simulated data we use a fully labelled dataset and simulate the performance of these approaches to confirm that we can reach the desired recall threshold of 95%. The authors also recommend building safeguards into the process to reduce the number of Type 2 errors when possible, including supplementing the keyword search with handsearching, soliciting expert knowledge, and reviewing bibliographies of relevant preprints or recent articles.

One major strength of this study was the efficiency the automated approach afforded, and which other researchers can hopefully achieve by adopting similar approaches. Researchers have estimated that screening a title and abstract takes about two minutes of human labor across two screeners [29], meaning that excluding over 5,000 studies from manual review alone saved over 160 hours of researcher time. In the event screening is completed by paid research assistants, this may correspond to important budget impacts, a key consideration in the responsible stewardship of research funds. For example, the savings would be a minimum of $2,500 based on the standard hourly wage of $18–20 for Research Assistants at Northwestern University, where the study was conducted. The incorporation of human expertise was essential in the use of automated methods in this study; specifically, human experts guided feature selection, model development, and result validation and stratified some items for manual screening to optimize the use of automated tools. Overall, the timesaving achieved from application of the automated approach to screening was especially useful given that the search was low in precision, a challenge that other SGM researchers are also likely to encounter when conducting systematic reviews. Researchers in other areas or with narrower-scope research topics may achieve higher-precision results when using these methods.

Although the search was low precision even after prioritizing studies with machine learning, this was not unexpected given the nature of our constructed search strategy, as SGM health research has been expanding [30], searches of related topics have been similarly high-volume [31], and little attention has been paid to SOGI measurement relative to the total body of research on SGM health. The low-precision search does not undermine the utility of the automated approach, as without this approach, screening results would have been more resource-intensive. However, there is still significant room for improvement in precision when using automation to identify relevant literature. Large language models (LLMs) such as those incorporated into generative AI tools from Google, OpenAI, and Anthropic show potential for improving precision in the application of article screening [26].

In future work, researchers should consider applying machine learning tools to test these approaches in other areas of SGM health, COVID-19, HIV and other infectious diseases, and tobacco research to aid in identifying other contexts in which use of these methods might be most useful. Using a machine learning approach for future systematic reviews—and incorporating partnerships with experienced librarians when doing so—has the potential to ensure that researchers can efficiently search, review and synthesize the literature to make the most comprehensive and well-informed recommendations for future research and practice.

## Data Availability

The database search strategies are available in searchRxiv https://www.cabidigitallibrary.org/doi/10.1079/searchRxiv.2024.00566.

## SUPPLEMENTAL FILES

**Appendix A:** Recognize Methods
