# Supplementary material for: Automated tools for systematic review screening methods: an application of machine learning for sexual orientation and gender identity measurement in health research
Source: J Med Libr Assoc. 2025 Jan 14;113(1):31–8. doi: 10.5195/jmla.2025.1860 (PMC11835039; doi:10.5195/jmla.2025.1860)
Supplement: Supplementary file 1 — Appendix A: Recognize Methods [file jmla-113-1-31-s01.docx]

**Appendix. Full search strategies**

**Bibliographic databases searched and records produced**

| **Database** | **Results** | **Notes/Limits** |
| --- | --- | --- |
| PubMed | 7602 | Previous 10 years |
| CINAHL | 3489 | Previous 10 years |
| HAPI | 137 | Previous 10 years |
| PsycInfo | 6586 | Previous 10 years |
| **Total Results** | 17,814 | NA |
| **Duplicates Removed** | 6,373 | NA |
| **# of References Considered for Manual Screening** | 11,441 |  |

**Search strategy documentation**

All searches were run on June 7, 2022.

**PubMed via U.S. National Library of Medicine/National Institutes of Health**

| 1 | (instrument[tiab] OR instruments[tiab] OR Measurement[tiab] OR Measurements[tiab] OR Measures[tiab] OR Measure[tiab] OR scale[tiab] OR scales[tiab]OR validate[tiab] OR validation[tiab] OR validates[tiab] OR validated[tiab] OR validity[tiab]) |  |
| --- | --- | --- |
| 2 | (bisexual[tiab] OR "bisexuality"[mesh] OR bisexuality[tiab] OR bisexuals[tiab] OR "sexual and gender minorities"[mesh] OR "homosexuality, female"[mesh] OR homosexuality[mesh] OR "homosexuality, male"[mesh] OR "transgender persons"[mesh] OR (FTM[tiab] AND transgender[tiab]) OR (MTF[tiab] AND transgender[tiab]) OR ("male-to-female"[tiab] AND transgender[tiab]) OR ("female-to-male"[tiab] AND transgender[tiab]) OR gay[tiab] OR gays[tiab] OR "gender dysphoria"[tiab] OR "gender identity"[tiab] OR "gender reassign"[tiab] OR "gender reassignment"[tiab] OR "gender transition"[tiab] OR genderqueer[tiab] OR homosexual[tiab] OR homosexualities[tiab] OR homosexuality[tiab] OR homosexuals[tiab] OR intersex[tiab] OR lesbian[tiab] OR lesbians[tiab] OR "men who have sex with men"[tiab] OR (msm[tiab] AND sex[tiab]) OR queer[tiab] OR "same gender loving"[tiab] OR "same sex attracted"[tiab] OR "same sex couple"[tiab] OR "same sex couples"[tiab] OR "same sex relations"[tiab] OR "sexual and gender minorities"[tiab] OR "sexual and gender minority"[tiab] OR "sexual identity"[tiab] OR "sexual minorities"[tiab] OR "sexual minority"[tiab] OR "sexual orientation"[tiab] OR "sexual preference"[tiab] OR "sex characteristic"[tiab] OR "sex characteristics"[tiab] OR "sexual characteristic"[tiab]OR "sexual characteristics"[tiab] OR "sexual behavior"[tiab] OR "sexual attraction"[tiab]OR "sexual attractions"[tiab] OR "gender minority"[tiab] OR "gender minorities"[tiab] OR "gender expression*"[tiab] OR "gender diversity"[tiab] OR "gender diverse"[tiab] OR "gender nonbinary"[tiab] OR "gender non-binary"[tiab] OR "differences of sex development"[tiab] OR "disorders of sex development"[tiab] OR hermaphrodite[tiab] OR "gender non-conforming"[tiab] OR asexual[tiab] OR pansexual[tiab] OR queer[tiab] OR "gender modality"[tiab] OR "two-spirit"[tiab] OR "two spirits"[tiab] OR "same sex attraction"[tiab] OR androphilia[tiab] OR gynephilia[tiab] OR "trans female"[tiab] OR "trans male"[tiab] OR "trans man"[tiab] OR "trans men"[tiab] OR "trans people"[tiab] OR "trans person"[tiab] OR "trans woman"[tiab] OR transexuality[tiab] OR transexuals[tiab] OR transgender[tiab] OR transgendered[tiab] OR transgenders[tiab] OR transsexual[tiab] OR transsexualism[mesh] OR transsexualism[tiab] OR transsexuality[tiab] OR transsexuals[tiab] OR transvestite[tiab] OR "women loving women"[tiab] OR "women who have sex with women"[tiab] OR (WSW[tiab]AND sex)) |  |
| **3** | (gay[au] OR "markov state model"[tiab] OR "multiple source method"[tiab] OR "Modulation transfer function"[tiab]) |  |
| **4** | **1 AND (2 NOT 3)** |  |
| **5** | 4 AND (2012:2022[pdat]) |  |
| **6** | **5 NOT** ("animals"[MeSH] NOT "humans"[MeSH]) | **7607** |

**CINAHL via EBSCOhost**

| 1 | (((TI Measure OR AB Measure) OR (TI Measurement OR AB Measurement) OR (TI Measurements OR AB Measurements) OR (TI Measures OR AB Measures) OR (TI scale OR AB scale) OR (TI scales OR AB scales) OR (TI validation OR AB validation) OR (TI instrument OR AB instrument) OR (TI instruments OR AB instruments) OR (TI validate OR AB validate) OR (TI validation OR AB validation) OR (TI validates OR AB validates) OR (TI validated OR AB validated) OR (TI validity OR AB validity))) |  |
| --- | --- | --- |
| 2 | (((TI bisexual OR AB bisexual) OR (MH bisexuality) OR (TI bisexuality OR AB bisexuality) OR (TI bisexuals OR AB bisexuals) OR (MH "sexual and gender minorities+”) OR (MH homosexuality) OR (MH "Transgender Persons+") OR (MH "Asexuality") OR (MH “Bisexuality") OR ((TI FTM OR AB FTM) AND (TI transgender OR AB transgender)) OR ((TI MTF OR AB MTF) AND (TI transgender OR AB transgender)) OR ((TI male-to-female OR AB male-to-female) AND (TI transgender OR AB transgender)) OR ((TI female-to-male OR AB female-to-male) AND (TI transgender OR AB transgender)) OR (TI gay OR AB gay) OR (TI gays OR AB gays) OR (TI "gender dysphoria" OR AB "gender dysphoria") OR (TI "gender identity" OR AB "gender identity") OR (TI "gender reassign" OR AB "gender reassign") OR (TI "gender reassignment" OR AB "gender reassignment") OR (TI "gender transition" OR AB "gender transition") OR (TI genderqueer OR AB genderqueer) OR (TI homosexual OR AB homosexual) OR (TI homosexualities OR AB homosexualities) OR (TI homosexuality OR AB homosexuality) OR (TI homosexuals OR AB homosexuals) OR (TI intersex OR AB intersex) OR (TI lesbian OR AB lesbian) OR (TI lesbians OR AB lesbians) OR (TI "men who have sex with men" OR AB "men who have sex with men") OR ((TI msm OR AB msm) AND (TI sex OR AB sex)) OR (TI queer OR AB queer) OR (TI "same gender loving" OR AB "same gender loving") OR (TI "same sex attracted" OR AB "same sex attracted") OR (TI "same sex couple" OR AB "same sex couple") OR (TI "same sex couples" OR AB "same sex couples") OR (TI "same sex relations" OR AB "same sex relations") OR (TI "sexual and gender minorities" OR AB "sexual and gender minorities") OR (TI "sexual and gender minority" OR AB "sexual and gender minority") OR (TI "sexual identity" OR AB "sexual identity") OR (TI "sexual minorities" OR AB "sexual minorities") OR (TI "sexual minority" OR AB "sexual minority") OR (TI "sexual orientation" OR AB "sexual orientation") OR (TI "sexual preference" OR AB "sexual preference") OR (TI "sex characteristic" OR AB "sex characteristic") OR (TI "sex characteristics" OR AB "sex characteristics") OR (TI "sexual characteristic” OR "sexual characteristics” OR AB "sexual characteristic” OR "sexual characteristics”) OR (TI "sexual behavior" OR AB "sexual behavior") OR (TI "sexual attraction” OR "sexual attractions” OR AB "sexual attraction” OR "sexual attractions”) OR (TI "gender minority" OR AB "gender minority") OR (TI "gender minorities" OR AB "gender minorities") OR (TI "gender expression*" OR AB "gender expression*") OR (TI "gender diversity" OR AB "gender diversity") OR (TI "gender diverse" OR AB "gender diverse") OR (TI "gender nonbinary" OR AB "gender nonbinary") OR (TI "gender non-binary" OR AB "gender non-binary") OR (TI "differences of sex development" OR AB "differences of sex development") OR (TI "disorders of sex development" OR AB "disorders of sex development") OR (TI hermaphrodite OR AB hermaphrodite) OR (TI "gender non-conforming" OR AB "gender non-conforming") OR (TI asexual OR AB asexual) OR (TI pansexual OR AB pansexual) OR (TI queer OR AB queer) OR (TI "gender modality" OR AB "gender modality") OR (TI two-spirit OR AB two-spirit) OR (TI "two spirits" OR AB "two spirits") OR (TI "same sex attraction" OR AB "same sex attraction") OR (TI androphilia OR AB androphilia) OR (TI gynephilia OR AB gynephilia) OR (TI "trans female" OR AB "trans female") OR (TI "trans male" OR AB "trans male") OR (TI "trans man" OR AB "trans man") OR (TI "trans men" OR AB "trans men") OR (TI "trans people" OR AB "trans people") OR (TI "trans person" OR AB "trans person") OR (TI "trans woman" OR AB "trans woman") OR (TI transexuality OR AB transexuality) OR (TI transexuals OR AB transexuals) OR (TI transgender OR AB transgender) OR (TI transgendered OR AB transgendered) OR (TI transgenders OR AB transgenders) OR (TI transsexual OR AB transsexual) OR (TI transsexualism OR AB transsexualism) OR (TI transsexuality OR AB transsexuality) OR (TI transsexuals OR AB transsexuals) OR (TI transvestite OR AB transvestite) OR (TI "women loving women" OR AB "women loving women") OR (TI "women who have sex with women" OR AB "women who have sex with women") OR (TI "women having sex with women" OR AB "women having sex with women") OR (((TI "WSW” OR AB "WSW”) AND (TI sex OR AB sex))) |  |
| 3 | ( (TI "markov state model" OR AB "markov state model") OR (TI "multiple source method" OR AB "multiple source method") OR (TI "Modulation transfer function" OR AB "Modulation transfer function"))) |  |
| 4 | 1 AND 2 NOT 3 |  |
| 5 | 4 NOT ((MH animals+)) |  |
| 6 | Filter applied: Published date 20120101- | **3487** |

**PsycInfo via EBSCOhost**

| 1 | (((TI Measure OR AB Measure) OR (TI Measurement OR AB Measurement) OR (TI Measurements OR AB Measurements) OR (TI Measures OR AB Measures) OR (TI scale OR AB scale) OR (TI scales OR AB scales) OR (TI instrument OR AB instrument) OR (TI instruments OR AB instruments) OR (TI validate OR AB validate) OR (TI validation OR AB validation) OR (TI validates OR AB validates) OR (TI validated OR AB validated) OR (TI validity OR AB validity) OR (DE “Measurement”) )) |  |
| --- | --- | --- |
| 2 | (((TI bisexual OR AB bisexual) OR (TI bisexuality OR AB bisexuality) OR (TI bisexuals OR AB bisexuals) OR (DE “Bisexuality") OR (DE "Sexual Minority Groups") OR (DE "LGBTQ") OR (DE "Homosexuality") OR (DE "Transgender") OR (DE "Transsexualism") OR (DE "Asexuality") OR (DE "Lesbianism") OR (DE "Male Homosexuality") OR ((TI FTM OR AB FTM) AND (TI transgender OR AB transgender)) OR ((TI MTF OR AB MTF) AND (TI transgender OR AB transgender)) OR ((TI male-to-female OR AB male-to-female) AND (TI transgender OR AB transgender)) OR ((TI female-to-male OR AB female-to-male) AND (TI transgender OR AB transgender)) OR (TI gay OR AB gay) OR (TI gays OR AB gays) OR (TI "gender dysphoria" OR AB "gender dysphoria") OR (TI "gender identity" OR AB "gender identity") OR (TI "gender reassign" OR AB "gender reassign") OR (TI "gender reassignment" OR AB "gender reassignment") OR (TI "gender transition" OR AB "gender transition") OR (DE "Gender Transition") OR (DE "Gender Identity") OR (DE "Gender Nonconforming") OR (DE "Gender Nonbinary") OR (TI genderqueer OR AB genderqueer) OR (TI homosexual OR AB homosexual) OR (TI homosexualities OR AB homosexualities) OR (TI homosexuality OR AB homosexuality) OR (TI homosexuals OR AB homosexuals) OR (TI intersex OR AB intersex) OR (DE “intersex”) OR (TI lesbian OR AB lesbian) OR (TI lesbians OR AB lesbians) OR (TI "men who have sex with men" OR AB "men who have sex with men") OR ((TI msm OR AB msm) AND (TI sex OR AB sex)) OR (TI queer OR AB queer) OR (TI "same gender loving" OR AB "same gender loving") OR (TI "same sex attracted" OR AB "same sex attracted") OR (TI "same sex couple" OR AB "same sex couple") OR (TI "same sex couples" OR AB "same sex couples") OR (TI "same sex relations" OR AB "same sex relations") OR (DE "Sex and Gender Measures") OR(TI "sexual and gender minorities" OR AB "sexual and gender minorities") OR (TI "sexual and gender minority" OR AB "sexual and gender minority") OR (TI "sexual identity" OR AB "sexual identity") OR (TI "sexual minorities" OR AB "sexual minorities") OR (TI "sexual minority" OR AB "sexual minority") OR (TI "sexual orientation" OR AB "sexual orientation") OR (TI "sexual preference" OR AB "sexual preference") OR (TI "sex characteristic" OR AB "sex characteristic") OR (TI "sex characteristics" OR AB "sex characteristics") OR (TI "sexual characteristic” OR "sexual characteristics” OR AB "sexual characteristic” OR "sexual characteristics”) OR (TI "sexual behavior" OR AB "sexual behavior") OR (TI "sexual attraction” OR "sexual attractions” OR AB "sexual attraction” OR "sexual attractions”) OR (TI "gender minority" OR AB "gender minority") OR (TI "gender minorities" OR AB "gender minorities") OR (TI "gender expression*" OR AB "gender expression*") OR (TI "gender diversity" OR AB "gender diversity") OR (TI "gender diverse" OR AB "gender diverse") OR (TI "gender nonbinary" OR AB "gender nonbinary") OR (TI "gender non-binary" OR AB "gender non-binary") OR (TI "differences of sex development" OR AB "differences of sex development") OR (TI "disorders of sex development" OR AB "disorders of sex development") OR (TI hermaphrodite OR AB hermaphrodite) OR (TI "gender non-conforming" OR AB "gender non-conforming") OR (TI asexual OR AB asexual) OR (TI pansexual OR AB pansexual) OR (TI queer OR AB queer) OR (TI "gender modality" OR AB "gender modality") OR (TI two-spirit OR AB two-spirit) OR (TI "two spirits" OR AB "two spirits") OR (TI "same sex attraction" OR AB "same sex attraction") OR (TI androphilia OR AB androphilia) OR (TI gynephilia OR AB gynephilia) OR (TI "trans female" OR AB "trans female") OR (TI "trans male" OR AB "trans male") OR (TI "trans man" OR AB "trans man") OR (TI "trans men" OR AB "trans men") OR (TI "trans people" OR AB "trans people") OR (TI "trans person" OR AB "trans person") OR (TI "trans woman" OR AB "trans woman") OR (TI transexuality OR AB transexuality) OR (TI transexuals OR AB transexuals) OR (TI transgender OR AB transgender) OR (TI transgendered OR AB transgendered) OR (TI transgenders OR AB transgenders) OR (TI transsexual OR AB transsexual) OR (TI transsexualism OR AB transsexualism) OR (TI transsexuality OR AB transsexuality) OR (TI transsexuals OR AB transsexuals) OR (TI transvestite OR AB transvestite) OR (TI "women loving women" OR AB "women loving women") OR (TI "women who have sex with women" OR AB "women who have sex with women") OR (TI "women having sex with women" OR AB "women having sex with women") OR (((TI "WSW” OR AB "WSW”) AND (TI sex OR AB sex))) |  |
| 3 | ( (TI "markov state model" OR AB "markov state model") OR (TI "multiple source method" OR AB "multiple source method") OR (TI "Modulation transfer function" OR AB "Modulation transfer function"))) |  |
| 4 | 1 AND 2 NOT 3 |  |
| 5 | Filter applied: Published date 20120101- | 6,571 |

**Health and Psychosocial Instruments (HaPI) via EBSCOhost**

| 1 | (((TI bisexual OR AB bisexual) OR (TI bisexuality OR AB bisexuality) OR (TI bisexuals OR AB bisexuals) OR (ZU "bisexuality") OR (TI "sexual and gender minorities” OR AB "sexual and gender minorities”) OR (ZU "homosexuality") OR (ZU "transexualism") or (ZU "transgender") or (ZU "transgender persons") OR (TI "Asexuality" OR AB “asexuality”) OR ((TI FTM OR AB FTM) AND (TI transgender OR AB transgender)) OR ((TI MTF OR AB MTF) AND (TI transgender OR AB transgender)) OR ((TI male-to-female OR AB male-to-female) AND (TI transgender OR AB transgender)) OR ((TI female-to-male OR AB female-to-male) AND (TI transgender OR AB transgender)) OR (TI gay OR AB gay) OR (TI gays OR AB gays) OR (TI "gender dysphoria" OR AB "gender dysphoria") OR (TI "gender identity" OR AB "gender identity") OR (TI "gender reassign" OR AB "gender reassign") OR (TI "gender reassignment" OR AB "gender reassignment") OR (TI "gender transition" OR AB "gender transition") OR (TI genderqueer OR AB genderqueer) OR (TI homosexual OR AB homosexual) OR (TI homosexualities OR AB homosexualities) OR (TI homosexuality OR AB homosexuality) OR (TI homosexuals OR AB homosexuals) OR (TI intersex OR AB intersex) OR (TI lesbian OR AB lesbian) OR (TI lesbians OR AB lesbians) OR (TI "men who have sex with men" OR AB "men who have sex with men") OR ((TI msm OR AB msm) AND (TI sex OR AB sex)) OR (TI queer OR AB queer) OR (TI "same gender loving" OR AB "same gender loving") OR (TI "same sex attracted" OR AB "same sex attracted") OR (TI "same sex couple" OR AB "same sex couple") OR (TI "same sex couples" OR AB "same sex couples") OR (TI "same sex relations" OR AB "same sex relations") OR (TI "sexual and gender minorities" OR AB "sexual and gender minorities") OR (TI "sexual and gender minority" OR AB "sexual and gender minority") OR (TI "sexual identity" OR AB "sexual identity") OR (TI "sexual minorities" OR AB "sexual minorities") OR (TI "sexual minority" OR AB "sexual minority") OR (TI "sexual orientation" OR AB "sexual orientation") OR (TI "sexual preference" OR AB "sexual preference") OR (TI "sex characteristic" OR AB "sex characteristic") OR (TI "sex characteristics" OR AB "sex characteristics") OR (TI "sexual characteristic” OR "sexual characteristics” OR AB "sexual characteristic” OR "sexual characteristics”) OR (TI "sexual behavior" OR AB "sexual behavior") OR (TI "sexual attraction” OR "sexual attractions” OR AB "sexual attraction” OR "sexual attractions”) OR (TI "gender minority" OR AB "gender minority") OR (TI "gender minorities" OR AB "gender minorities") OR (TI "gender expression*" OR AB "gender expression*") OR (TI "gender diversity" OR AB "gender diversity") OR (TI "gender diverse" OR AB "gender diverse") OR (TI "gender nonbinary" OR AB "gender nonbinary") OR (TI "gender non-binary" OR AB "gender non-binary") OR (TI "differences of sex development" OR AB "differences of sex development") OR (TI "disorders of sex development" OR AB "disorders of sex development") OR (TI hermaphrodite OR AB hermaphrodite) OR (TI "gender non-conforming" OR AB "gender non-conforming") OR (ZU "gender nonconforming") OR (TI asexual OR AB asexual) OR (TI pansexual OR AB pansexual) OR (TI queer OR AB queer) OR (TI "gender modality" OR AB "gender modality") OR (TI two-spirit OR AB two-spirit) OR (TI "two spirits" OR AB "two spirits") OR (TI "same sex attraction" OR AB "same sex attraction") OR (TI androphilia OR AB androphilia) OR (TI gynephilia OR AB gynephilia) OR (TI "trans female" OR AB "trans female") OR (TI "trans male" OR AB "trans male") OR (TI "trans man" OR AB "trans man") OR (TI "trans men" OR AB "trans men") OR (TI "trans people" OR AB "trans people") OR (TI "trans person" OR AB "trans person") OR (TI "trans woman" OR AB "trans woman") OR (TI transexuality OR AB transexuality) OR (TI transexuals OR AB transexuals) OR (TI transgender OR AB transgender) OR (TI transgendered OR AB transgendered) OR (TI transgenders OR AB transgenders) OR (TI transsexual OR AB transsexual) OR (TI transsexualism OR AB transsexualism) OR (TI transsexuality OR AB transsexuality) OR (TI transsexuals OR AB transsexuals) OR (TI transvestite OR AB transvestite) OR (TI "women loving women" OR AB "women loving women") OR (TI "women who have sex with women" OR AB "women who have sex with women") OR (TI "women having sex with women" OR AB "women having sex with women") OR (((TI "WSW” OR AB "WSW”) AND (TI sex OR AB sex))) |  |
| --- | --- | --- |
| 2 | Filter applied: Published date 20120101- | 137 |
